# Femtosecond Laser-Micromachining of Glass Micro-Chip for High Order Harmonic Generation in Gases

**DOI:** 10.3390/mi11020165

**Published:** 2020-02-04

**Authors:** Anna G. Ciriolo, Rebeca Martínez Vázquez, Alice Roversi, Aldo Frezzotti, Caterina Vozzi, Roberto Osellame, Salvatore Stagira

**Affiliations:** 1 Institute for Photonics and Nanotechnologies, National Research Council, 20133 Milan, Italy; annagabriella.ciriolo@polimi.it (A.G.C.); caterina.vozzi@polimi.it (C.V.); roberto.osellame@polimi.it (R.O.); salvatore.stagira@polimi.it (S.S.); 2 Department of Physics, Politecnico di Milano, 20133 Milan, Italy; alice.rov@libero.it; 3 Department of Aerospace Science and Technology, Politecnico di Milano, 20156 Milan, Italy; aldo.frezzotti@polimi.it

**Keywords:** femtosecond laser micromachining, high order harmonic generation, de laval gas micro nozzle, attosecond science

## Abstract

We report on the application of femtosecond laser micromachining to the fabrication of complex glass microdevices, for high-order harmonic generation in gas. The three-dimensional capabilities and extreme flexibility of femtosecond laser micromachining allow us to achieve accurate control of gas density inside the micrometer interaction channel. This device gives a considerable increase in harmonics’ generation efficiency if compared with traditional harmonic generation in gas jets. We propose different chip geometries that allow the control of the gas density and driving field intensity inside the interaction channel to achieve quasi phase-matching conditions in the harmonic generation process. We believe that these glass micro-devices will pave the way to future downscaling of high-order harmonic generation beamlines.

## 1. Introduction

Since its first observation more than twenty years ago, it has been known that an intense and ultrashort laser pulse, focused in a gaseous medium, drives the emission of a burst of coherent radiation, collinear to the driving beam, with a spectral content ranging from the vacuum ultraviolet to the soft X rays [1]. This process is called high-order harmonic generation (HHG) since the spectrum of the emitted radiation appears as a combination of numerous odd harmonics of the fundamental laser field. In the temporal domain, this emission is structured as a train of attosecond light pulses, and indeed, HHG is routinely exploited in the fields of extreme ultraviolet spectroscopy and Attosecond Science [2]. 

The manipulation of HHG beams is done in grazing incidence on bulky and expensive optics that require careful alignment and even active stabilization systems, and, as a consequence, these beam lines extend over several meters, occupying entire rooms. Up to now, HHG-based eXtreme Ultra Violet (XUV, from 100 nm down to 10 nm) and soft X (from 10 nm down to 1 nm) coherent light sources are confined within a few advanced laboratories [3] because of their technological complexity. In this framework, a substantial breakthrough in ultrafast technology can be achieved by the miniaturization of HHG beamlines, which could remarkably foster the application of HHG sources in numerous novel fields. 

A strategy to obtain such compact sources is to generate high-order harmonics in gas-filled glass capillaries of a few centimeters length, which behave as hollow waveguides [4,5,6]. Hollow waveguides are already widely exploited in the field of ultrafast laser sources mainly for the compression of intense pulses [6], but also in the field of HHG, they lead to impressive results allowing hundreds times enhancement of the intensity of the harmonics to be reached [4] and spectral components up to the keV photon energy [5] in tabletop systems. 

A route for achieving even higher miniaturization is based on femtosecond laser micromachining, which is a powerful fabrication technique that has already demonstrated its high potential in the fabrication of miniaturized lab-on-chip devices [7]. Ultrashort laser pulses are focused inside a transparent sample and, due to nonlinear absorption processes, such as multiphoton and avalanche ionization, they produce a permanent modification of the material only at a small region around the focus where the highest intensity is reached [8,9].

The photogenerated hot-electron plasma during fs-laser irradiation induces high temperatures and pressures that give rise to different phenomena, such as densification, direct photo structural modifications, and color-center formation. Under suitable conditions, the combination of such effects may lead to a local increase in the etching speed of the material over a micrometer-sized volume [10]. By moving the laser focus inside the substrate, one can use the laser beam to define three-dimensional regions of increased etching speed. A powerful method for the fabrication of microfluidic networks, in a 3D geometry, directly buried in the glass substrate is femtosecond laser irradiation of fused silica glasses followed by chemical etching (FLICE). Until now, the devices fabricated by FLICE have been extensively used for the manipulation of liquids, but they are perfectly suitable for the manipulation of gases as well.

In this work, we will present gas-filled microfluidic devices, fabricated through the FLICE technique, demonstrating efficient emission of extreme ultraviolet radiation produced by HHG. We exploit the flexibility, accuracy, and 3D capabilities of the FLICE technique to create glass micro-devices for manipulating gas fluxes and for guiding laser beams through microchannels. 

## 2. Materials and Methods 

**Figure 1:**
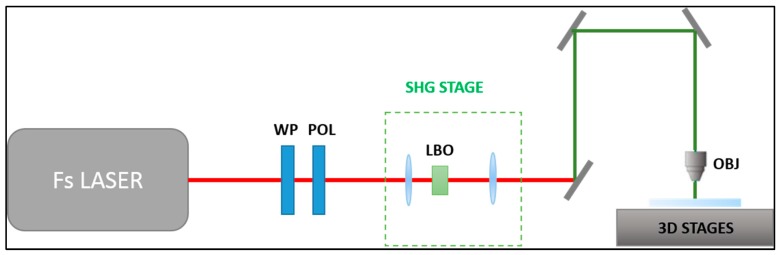
Femtosecond laser micromachining setup. The laser beam passed through an attenuation module, made of a λ/2 waveplate and a polarizer, and then through a telescope and a non-linear crystal (BBO) for second harmonic (515 nm) generation; the second harmonic beam was focused onto the glass sample. The sample was mounted on to a 3-dimension translation stage.

For the femtosecond laser micromachining of the glass devices, the second harmonic (515 nm) of a femtosecond laser beam (Satsuma, Amplitude Systemes S.A., Pessac, France) was focused inside a fused silica sample using a 63 × (0.65 NA) microscope objective (LD-plan Neofluar, Zeiss, Oberkochen, Germany). As represented in the scheme of Figure 1, the glass sample was mounted onto a high-resolution 3D movement system (Aerotech, ANT, Hampshire, UK) and moved with respect to the laser beam following the desired trajectory. The laser repetition rate was set at 1 MHz, with a pulse duration of 230 fs and pulse energy between 200 nJ and 300 nJ depending on the dimensions of the irradiated geometry. After the laser irradiation, the sample was immersed in an ultrasonic bath of a 20% HF aqueous solution at 35 °C. 

**Figure 2:**
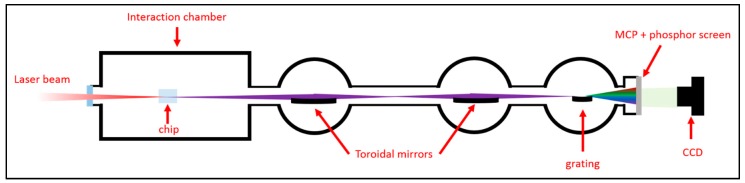
High-order harmonics generation and acquisition setup. The chip was mounted onto a home-built 5-axis translation stage to allow a precise coupling of the driving laser.

HHG experiments were performed under vacuum conditions in a beamline composed of an interaction chamber (working pressure 10^−5^ mbar) and a grazing incidence XUV spectrometer (working pressure 10^−6^–10^−7^ mbar) (see Figure 2). The beam of a Ti:Sapphire laser (25 fs, 1 kHz, 10 mJ) was coupled into the microchannel, which was located inside the interaction chamber. The driving laser beam was focused at the entrance of the hollow waveguide by a 20-cm focal lens. The spatial coupling of the beam with the microchannel was achieved with the help of a high-precision motorized alignment stage. The gas pressure was accurately controlled by a system composed of a needle valve and a pressure gauge placed in the gas line after the needle valve. The high-order harmonics radiation was acquired by a spectrometer composed of grazing-incidence optics. A first toroidal mirror was used for generating an intermediate focus for spectroscopy purposes. The second toroidal mirror focused the HHG beam on a dispersion grating. The dispersed HHG signal was acquired by means of a vacuum compatible detector (Photek, VID140, East Sussex, UK) incorporating one micro-channel plate (MCP) and a P20 phosphor screen. The image displayed on the phosphor screen was acquired by a CCD camera (Apogee Ascent A2150, Andor, Belfast, UK). The same chip was used for HHG for several weeks, with no evident degradation.

We used Comsol Multiphysics software to model the gas speed, temperature, and density (in the steady-state) along the hollow waveguide. We applied the high Mach number flow model coupled with the so-called k-ϵ turbulence model in order to properly describe the turbulent flow behavior of the gas inside the device. For the discretization of the geometry, we used a triangular mesh, optimized by the software for the physics of the problem.

## 3. Results and Discussion

**Figure 3:**
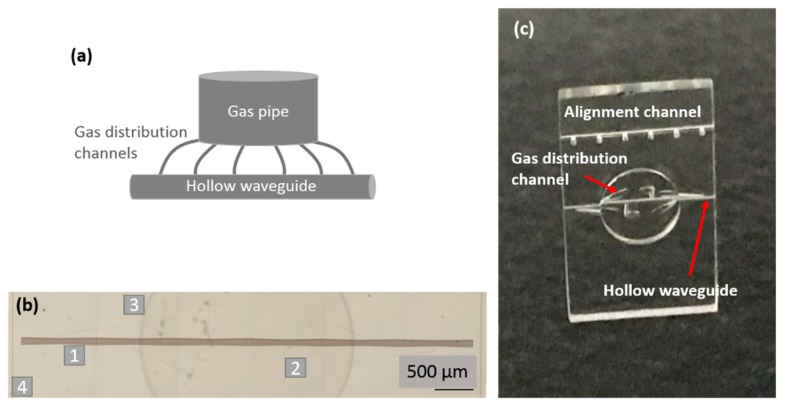
(**a**) Scheme of the high-order harmonic generation (HHG) glass chip, (**b**) microscope image of the irradiated sample, the different components are evidenced: 1-hollow waveguide, 2-gas redistribution channels, 3-gas pipe reservoir, 4-lateral cutting walls, (**c**) picture of the device after the HF etching.

The basic design of the glass chip for HHG is shown in Figure 3a; it consisted of a cylindrical top central reservoir (gas pipe) from which several small microchannels (gas distribution channels) departed and reached the cylindrical horizontal main channel (hollow waveguide). The gas was inserted through the top reservoir and, thanks to the homogeneously distributed small microchannels, it uniformly filled the hollow waveguide. In fact, the driving laser that propagated inside the waveguide encountered a uniform gas density. 

The final device was made in a fused silica plate (dimensions 6 × 10 × 1 mm^3^) and contained two parallel microchannels; the upper one served as an auxiliary channel for beam alignment, whereas the lower one was devoted to HHG. The reservoir for gas input had a radius of 1.6 mm, the small channels for gas distribution had a variable diameter from ~100 μm (at the reservoir base) to ~30 μm at the main channel surface. The main channel, which acted as a hollow waveguide, had a 120 μm diameter. Figure 3b,c show, respectively, the microscope image of the device after laser irradiation and a picture after chemical etching. The in-volume irradiated paths, which look darker in Figure 3b, led to embedded and interconnected empty channels after etching. The irradiated central channel presented a sinusoidal radius to compensate for inhomogeneous exposition to acid during the etching process, and to obtain a central channel with a constant radius after the etching step (see Figure 3c). It is important to point out that the FLICE technique allows combining structures with millimeter size (gas reservoir with 3 mm diameter) and small structures with micrometer size (microchannels) in the same device with just one fabrication process.

**Figure 4:**
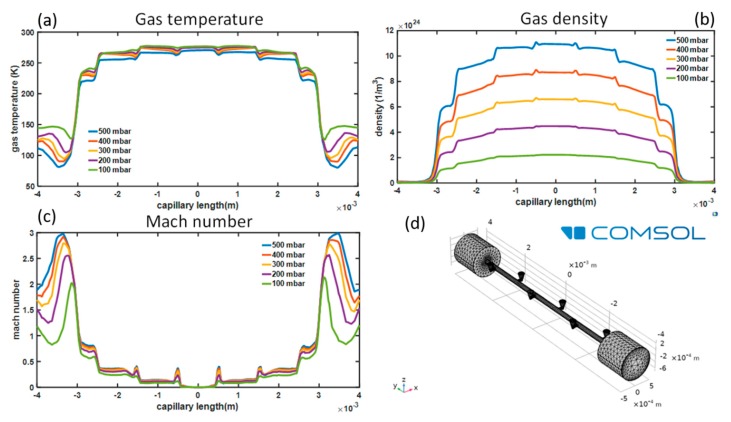
Numerical simulation of the Ne gas evolution inside the micromachined device reported in Figure 2, showing (**a**) gas temperature, (**b**) density, and (**c**) Mach number as a function of the gas backing pressure. (**d**) Gas volume geometry and mesh. The main channel and the small lateral features reproduce the internal chip structure. The two cylindrical structures placed at the extremities were used as output volume for simulating gas expansion in the interaction chamber. The simulations were performed using COMSOL.

To investigate the gas distribution in the microchip, we modeled the gas flow through the device in the final steady-state using Comsol Multiphysics. The mesh cell dimensions varied between a maximum value of 140 μm and a minimum value of 15 μm. The results, depending on the gas backing pressure, are shown in Figure 4. In particular, the gas temperature, density, and Mach number along the capillary axis are reported. With the exception of small peaks next to the outlets of the distribution channels, the gas flow exhibited the typical features of gas flows within micro-channels with a high length to diameter ratio. The gas flow was nearly isothermal and subsonic in most of the flow region. The density profile smoothly decreased from the center towards the waveguide outlets at the lowest backing pressure value, but it showed stronger spatial modulation at higher pressures. Abrupt changes in the behavior of all flow properties were observed in correspondence to the exits towards the vacuum chamber, where the gas underwent strong expansion.

The gas density inside the channel scaled linearly with the backing pressure. Thus, by properly monitoring the backing pressure, it is possible to achieve accurate control of the density of the generating medium inside the microchannel and, as a consequence, of the generated harmonics intensity.

**Figure 5:**
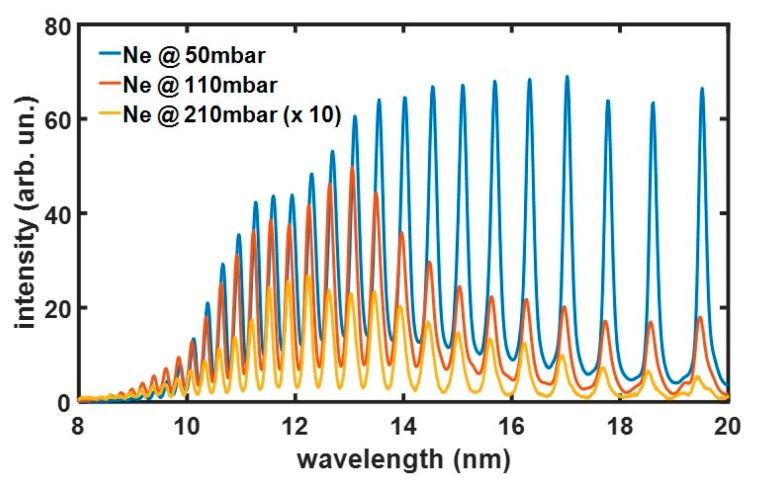
HHG spectra generated inside the chip using Ne gas with a backing pressure of 50 (blue), 110 (red), and 210 mbar (yellow); this latter curve is magnified 10 times.

For the HHG experiments, the gas pipe was connected to the glass chip, which was positioned into the interaction chamber, and a fraction of the laser beam (400 μJ pulse energy) was coupled inside the hollow waveguide. Figure 5 shows a comparison among different harmonic spectra generated inside the microchannel filled with neon gas. A decreasing yield was observed when the gas backing pressure was increased from 50 to 210 mbar. The reduction is more evident for low-order harmonics, as indicated by the reshaping of the harmonic spectrum. This pressure dependence is due to both phase-matching effects and absorption from the gas: as the pressure increases, the XUV radiation is strongly absorbed; moreover, the phase mismatch between the fundamental and the harmonic field worsens and leads to a dramatic reduction in the yield mainly in the low-energy part of the spectrum [11,12].

**Figure 6:**
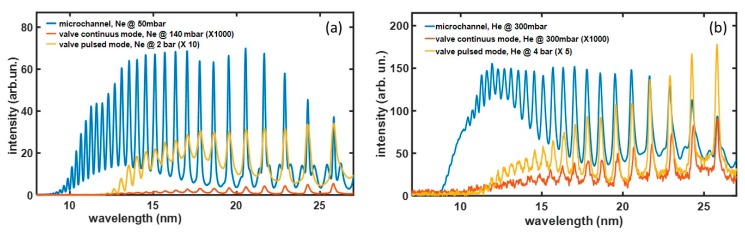
Comparison between HHG spectra produced inside the microchannel (blue), in a continuous gas jet (red), and in a pulsed gas jet (yellow) for Ne (**a**) and He (**b**) gases.

We compared the optimal HHG yield inside the gas-filled channel with that achieved in the most commonly used interaction geometry based on a gas jet. Figure 6 shows single-shot harmonics spectra generated inside the channel filled with neon at a 50-mbar backing pressure (a) or helium at a 300-mbar backing pressure (b). The results are compared to HHG in a gas jet produced by a 1-mm diameter valve (Parker) that is routinely used for gas-pulse generation in HHG, and that can be operated both in a steady and a pulsed mode. In the steady regime (red spectra), the valve was feed with gas pressures comparable to that used in the channel; in particular, 140 mbar of Neon and 300 mbar of Helium were used. In the pulsed regime (yellow spectra), higher backing pressures, on the multi-bar scale, were used for reproducing the standard working conditions of HHG experiments. Both with Helium and Neon, an extended cutoff, up to 7 nm (160 eV) was obtained inside the microchannel, whereas a cutoff of about 11 nm (110 eV) was observed in the jets. Moreover, an estimation of the harmonic generation yield in the spectral region detected by the spectrometer can be performed by the integration of the HHG spectra. As a result, a higher generation yield was achieved in the microchannel for both gases, which is up to 20 times that achieved with the pulsed jet and about 10^4^ times that obtained with the steady jet. The improved performances achieved in the microchannel are related both to the extension of the interaction region and the different interaction geometry with respect to the gas jets.

Although there is an overall improvement in the generation yield with respect to traditional HHG in gas, we are confident that a further improvement in the harmonic generation can be obtained by overcoming the phase-matching limitations. In fact, as in many nonlinear processes, HHG efficiency depends both on the single-atom response [13] and on the macroscopic response during propagation in the medium [14], so for generating a bright output beam the emission of a large number of atoms over an extended region must add in phase. The phase relationship between the fundamental and the harmonics field depends on several factors that are mainly related to the generating medium and generation geometry. In particular, the gas neutral and free-electron plasma optical dispersion is wavelength-dependent, so that the fundamental field and the newly generated harmonics can accumulate a phase-mismatch during the propagation. The geometrical phase effects depend instead on the space-structure of the fundamental beam, either tight-focusing or waveguide-assisted confinement.

Practical approaches exploiting quasi phase-matching (QPM) are challenging [5]. In this sense, we propose prototyping different microchips for HHG endowed with multiple gas micro-jets, with the attempt of exploiting QPM. We follow two main strategies which are known to be effective in the improvement in the HHG yield, i.e., the modulation of the driving field intensity or the modulation of the gas density [15,16].

**Figure 7:**
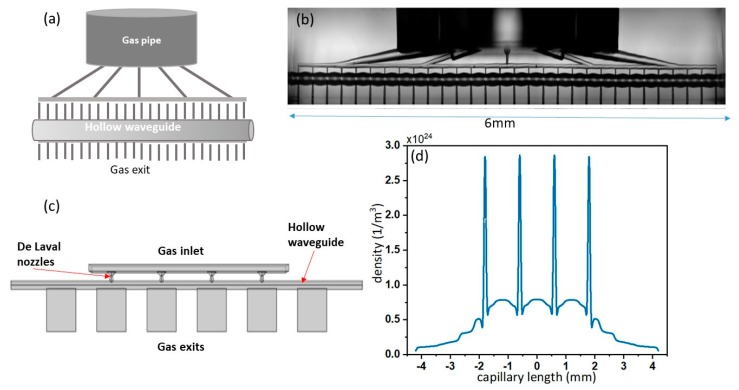
(**a**) Cartoon of the quasi phase-matching device with 32 gas inlets and modulated diameter. (**b**) Microscope image of the device in (**a**). (**c**) Cartoon of the device with four de Laval micro-nozzles for gas density periodic modulation. (**d**) Numerical simulation (Comsol) of gas density inside the chip shown in (**c**).

Modulation of the driving field intensity is achievable through modulation of the hollow waveguide diameter. In fact, the diameter of the propagating mode will depend on the waveguide diameter and as a consequence, also the peak intensity [17]. In Figure 7, panels (a) and (b) show the scheme and microscope image of the microchip with the modulated radius. The gas delivery inside the channel is similar to the one in the previous device; it is inserted through the top reservoir, flows through a network of microchannels, and reaches the hollow waveguides at some specific positions. In addition, in this device, each delivering microchannel faces a small exit channel in order to create a modulation of gas density. The strong capabilities of the FLICE technique are evidenced in this glass, which has a 20% radius modulation (110–135 μm) and 30 small gas inlets and outlets with micrometer size. 

It is possible to achieve an even higher modulation of the gas density inside the hollow waveguide by injecting the gas through a series of de Laval micro-nozzles whose supersonic flow condition, at the end of the divergent section, helps produce a more focused gas jet. The scheme of the chip is shown in Figure 7c. In this device, there is no gas reservoir, and the convergent section of each nozzle is almost directly facing the gas inlet. Different nozzle geometries were numerically investigated, using Comsol software, in order to enhance/control the density modulation along the axis of the hollow waveguide. 

It should be noticed that the nozzle flow behavior in the considered conditions deviates from the classical one, as described by the inviscid theory. Although the inlet pressure is high enough to avoid rarefaction effects, viscous forces are very pronounced (the nozzle throat Reynold’s number is about 300) and delay the supersonic transition within the divergent section whose effective area is strongly reduced by a thick boundary layer [18]. So far, the best nozzle configuration consists of a simple double cone geometry with an inlet diameter of 220 μm, a 60 μm throat diameter, and a 90 μm exit diameter, whose size is the largest as possible which preserves the guiding characteristics of the hollow waveguide. The overall nozzle length is 131 μm. Figure 7d shows the simulated gas density profile along the waveguide axis. In order to perform an accurate discretization of the problem, we used different meshes for the nozzle region, central waveguide, and gas exits. In particular, for the micro-nozzles, we used mesh cell dimensions that varied between a maximum element dimension of 5 μm and a minimum element dimension of 0.08 μm (near the walls). A strong density increase in correspondence with the nozzle position is clearly visible (~2 × 10^24^ molecules/m^3^). This density modulation is expected to be high enough to obtain QPM, given that the nozzle periodicity is the correct one.

## 4. Conclusions

In the present work, we demonstrated the application of femtosecond laser micromachined glass microchips to the generation of high-order harmonics in gas. The working principle relays in a main cylindrical microchannel that acts as a hollow waveguide where the driving laser propagates. This microchannel is filled with gas through a three-dimensional network of gas distribution channels all embedded in the glass device. The gas flow was numerically studied and characterized. As a result of the laser-gas interaction, an increase in the HHG signal was observed, which surpasses the performances of the standard generation configuration based on pulsed gas-jets both in terms of efficiency and spectral extension. 

By exploiting the extreme flexibility of the FLICE technique, different generation prototypes, including gas-micro-jets and periodically structured capillaries, were realized, aiming at further improving the harmonics generation yield in a quasi phase-matching regime. 

We foresee that the FLICE technique could be exploited to downscale an entire HHG-based beamline to a glass chip, integrating several additional functionalities, such as the separation of the IR driving beam from the HHG radiation by waveguide effects, and the inclusion of an interaction module for HHG spectroscopy in liquid and gaseous samples.
